# Concealed Gold Bridge Attachments

**Published:** 1901-04-15

**Authors:** H. M. Kirke


### CONCEALED GOLD BRIDGE ATTACHMENTS.

BY DR. H. M. KIRKE.

Number One.—"A method of bridge attachment designed to take the place of the shell crown, open-faced crown, or any other kind of crown, in the six anterior teeth, when entirely or comparatively free from caries."

This method is especially desirable in cases where all the central and lateral incisors are to be replaced, making the attachments to the cuspids. Devitalize the pulps and fill canals as usual. Bevel the lingual side of the tooth to be attached down to the incisive edge, allowing a liberal space for the thickness of the backing, in order to prevent the inferior incisors from impinging against it.

Take an impression of the prepared cuspid tooth in either plaster or mouldine and pour with fusible metal. Using this as a die, swage a backing of No. 30 gauge pure gold plate by driving the die into a soft pine block, cross section. Make the backing large enough to cover the entire lingual aspect of the tooth and overlapping well the edges, particularly on the mesial side. This backing may or may not extend underneath the gum, as is deemed best by the operator. Through this backing insert a platinum, or irido-platinum wire, gauge about 15; place on the tooth and allow the post to extend well up into the canal. Fasten the post and backing together with Parr's wax, remove, invest and solder slightly together with No. 22 carat or 20 carat solder; replace on tooth and burnish perfectly, especially around the edges. Adjust dummies, wax together, remove and invest again, and flow with 20 carat solder.

With a little finishing and re-burnishing, the attachment is ready for use. In this class of attachments, as in gold inlay fillings, the edge adaptation and burnishing are very important, and must not be neglected. Well nigh perfect edges may be obtained in this way, with proper manipulation of the pure gold base. Finish with stone and sandpaper discs and set with cement; when cement is set re-burnish all edges.

Number Two.—"A concealed gold attachment for short bridge or dummy; sound incisor tooth, living pulp."

Prepare porcelain tooth as in the preceding case, grinding and beveling to incisive edge enough to admit of strong backing. Drill four holes in lingual aspect of tooth large enough to admit a No. 20 gauge wire freely. Two of these holes are drilled toward the incisive edge of tooth, near the mesial and distal corners, and the other two toward the gum margin, and all four as nearly parallel as possible. The gingival pair of holes may be from one-eighth to three-sixteenths of an inch in length, the incisal pair considerably shallower. There will be little danger, if any, of coming into contact with the pulp if the drilling is near to the angles of the tooth; for it is well known that caries may be quite extensive on either the mesial or distal side of the tooth without involving the pulp. An impression of the lingual aspect of the tooth is then secured as before and poured up with fusible metal. Swage a backing of pure gold plate, 30 gauge, lapping well over the sides and incisive edge, and especially on the side next to the dummy space. Punch holes in the backing for the lower pair of holes, and insert short pins to the full length of the holes drilled in the tooth. This wire, as stated before, is irido-platinum, 20 gauge, with screw thread. Fit well and burnish and fasten backing and pins together with Parr's wax, invest and solder together with 20 carat solder. Replace the backing with the two pins in the tooth, punch the upper pair of holes, insert and tack the upper pair of pins as before. Fit again to the tooth and burnish perfectly, remove carefully, invest again and stiffen and thicken with 20 carat solder, when the backing will be ready to use as an attachment for a dummy or short bridge.

NUMBER THREE.—"Concealed bicuspid attachment for bridge, instead of shell or open-faced gold crown."

Devitalize the bicuspid, and prepare canal as usual through an opening drilled in the center of the sulcus ot the tooth. Enlarge for the accommodation of 20 gauge wire (irido-platinum) and of good length. With small carborundum stone increase the depth and breadth of the sulcus until it becomes a wide, deep groove, particularly so on the side adjoining the space for the dummy. If the dummies are to be inserted on the mesial side of the tooth, then this aspect of the tooth must be ground flat and perpendicular, straight up and down, from the occlusal edge of the tooth to a point slightly under the cervical margin. Take impression of tooth in plaster, make a fusible metal die, filling up the impressions of the adjoining teeth with mouldine, before pouring the metal. Swage 30 gauge pure gold over the mesial and occlusal aspects by driving the metal tooth into a transverse section into a block of soft pine wood. Punch hole for the 15 gauge irido-platinum post, place in position on the tooth with the post in the root canal, fasten with Parr's wax, remove, invest and tack post with 20 carat solder. Replace on the tooth and burnish and trim perfectly, allowing the plate to lap well over the edges. As in the preceding cases, a good fit and the subsequent durability of the bridge depend upon close adaptation of the gold backing, which can only be secured by burnishing and thickening with 20 carat solder as before. Solder on dummy, contour and finish properly, and a strong and artistic bridge pier is the result. Set with cement and finish on the tooth with carborundum stone and sandpaper discs.

